# Preparation and Characterization of New Nano-Composite Scaffolds Loaded With Vascular Stents

**DOI:** 10.3390/ijms13033366

**Published:** 2012-03-12

**Authors:** Hongzhen Xu, Jiansheng Su, Jun Sun, Tianbin Ren

**Affiliations:** 1 Institute of Prosthodontics, School of Stomatology, Tongji University, 399 Yanchang Road, Shanghai 200092, China; E-Mails: xuhongzhen0531@163.com (H.X.); cipher44444444@hotmail.com (J.S.); 2 Institute of Nano- and Bio-Polymeric Materials, School of Materials Science and Engineering, Tongji University, 1239 Siping Road, Shanghai 200092, China; E-Mail: rtb002@163.com

**Keywords:** nano-composite scaffold, tissue engineering, mechanical property, electrospinning

## Abstract

In this study, vascular stents were fabricated from poly (lactide-ɛ-caprolactone)/collagen/nano-hydroxyapatite (PLCL/Col/nHA) by electrospinning, and the surface morphology and breaking strength were observed or measured through scanning electron microscopy and tensile tests. The anti-clotting properties of stents were evaluated for anticoagulation surfaces modified by the electrostatic layer-by-layer self-assembly technique. In addition, nano-composite scaffolds of poly (lactic-co-glycolic acid)/polycaprolactone/nano-hydroxyapatite (PLGA/PCL/nHA) loaded with the vascular stents were prepared by thermoforming-particle leaching and their basic performance and osteogenesis were tested *in vitro* and *in vivo*. The results show that the PLCL/Col/nHA stents and PLGA/PCL/nHA nano-composite scaffolds had good surface structures, mechanical properties, biocompatibility and could guide bone regeneration. These may provide a new way to build vascularized-tissue engineered bone to repair large bone defects in bone tissue engineering.

## 1. Introduction

Bone tissue engineering has provided a new way to repair, restore or regenerate large bone defects impaired by disease, injury, or age [1]. Constructs are typically created by seeding a scaffold with cells *in vitro*. Scaffolds are an important component in bone tissue engineering and these should be three-dimensional, with a highly porous network of interconnected pores that can promote cell growth, adhesion, proliferation and differentiation by facilitating the flow and transport of nutrients and metabolic waste. In addition, they should have good biocompatibility and biodegradability to ensure they are non-toxic, non-allergic and cause no other adverse reactions to cells and the body and promote the formation of new tissues matched to the degradation rate of stent materials. Furthermore, they should have good biological activity to improve cell viability and promote tissue regeneration. Finally, good mechanical properties are also essential requirements of an ideal scaffold in bone tissue engineering [2–5]. Currently, vascularization has been the greatest problem in the successful repair of large bone defects by bone tissue engineering [1,6]. Therefore, the construction and mechanical, biological and anti-clotting properties of scaffolds are of crucial importance.

The normal vascular anatomy, in addition to capillaries and lymphatic capillaries, includes an outer membrane, a membrane and intimal layers. Endometrial lining is constituted by a single layer of epithelial cells in the basement membrane, and presents a smooth surface, rich in various types of collagen and elastin. The membrane is the thickest layer, with good mechanical properties, composed of smooth muscle cells arranged in layers of interaction. The adventitia is composed of loose connective tissue, which mainly contains fibroblast and type I collagen [7]. Ideal vascular stents should have the following characteristics: (1) they simulate the structure of normal vascular anatomy; (2) they have good biocompatibility and biological characteristics; (3) they have sufficient mechanical properties and compliance [8–10].

Currently, electrospinning has been the main method for tissue engineering of stents in many nano-fiber preparation technologies [11,12]. It has the following major advantages: (1) electrospun fiber stents have good biocompatibility; (2) precursor solutions of electrospinning are diverse; (3) the electrospinning process is highly controllable [13]. Nano-fibrous tissue engineered blood vessel stents have special advantages, as they are not only able to simulate the anatomy of the normal vascular tissue, but can mimic the composition and structure of the extracellular matrix to provide an ideal growing environment for cells, which are important prerequisites for the regeneration and reconstruction of blood vessels. In previous studies, tissue engineering of vascular stents could not fully be taken advantage of because implanting them into the body was prone to rapid thrombopoiesis, which is one of the limiting factors in clinical applications. To overcome this shortcoming, various methods for surface modification and functional modification on the stent surface exist that can improve performance by loading the surface with anti-clotting substances [14,15]. Layer-by-layer assembly has been one of the common means of modifying the stent surface in tissue engineering, which is not only suitable for materials with a variety of complex structures but also can fix a variety of biological molecules on the surface [16].

Collagen is the most abundant protein in the human body and a key element of the extracellular matrix (ECM) which imparts structural integrity and tensile strength to tissues. Recently, it has been used in a lot of tissue engineering applications as its predominance in the ECM, non-immunogenicity and available methods of isolation from a variety of sources. In addition, collagen fibers also possess some unique structural properties important for tissue engineering: they transmit forces, dissipate energy, prevent premature mechanical failure and provide biological signals to adjacent cells that regulate functional responses [17]. Collagen is resorbable, has high hydrophilicity, low antigenicity, very good biocompatibility and can promote tissue regeneration [18]. As it has many advantages, it has been one of the most ideal biopolymers available for tissue engineering applications.

Biodegradable polyesters, such as polylactic acid (PLA), polyglycolic acid (PGA) and their copolymers poly (lactic-co-glycolic acid) (PLGA), have good biocompatibility and biodegradation and are approved as a class of biological materials by the United States Food and Drug Administration (FDA). These show good mechanical strength, elastic modulus and can be thermally formed, so meet the basic requirements of carrier materials in bone and cartilage tissue engineering [19–21]. The mechanical properties and degradation rate of PLGA/PCL composite scaffolds can be improved by adjusting the molecular weight, structure and composition to meet different clinical requirements, and their ultimate degradation products are CO_2_ and H_2_O. Intermediate products of lactic acid are also the body’s normal sugar, so PLGA/PCL scaffold degradation *in vivo* will not have adverse effects on the organism [19,22,23]. In addition, excellent performances of composite materials have been widely reported in recent years [3,24]. The introduction of inorganic materials such as hydroxyapatite, calcium silicate not only enhance the mechanical strength of scaffold materials but can neutralize the acid produced by polymer degradation to maintain a stable pH value [25,26]. Synthetic hydroxyapatite, a type of inorganic material, has been extensively studied and applied, and is similar to natural bone. It has good biocompatibility, bioactivity and bone leading properties, and can guide new bone regeneration from the host bone along implant interfaces or internal implants to form a good bone combination [27–30].

In this study, electrospinning technology was applied to prepare small, biomimetic vascular stents of poly (lactide-ɛ-caprolactone)/collagen/nano-hydroxyapatite (PLCL/Col/nHA), and a modified layer of anti-coagulant which was built on the surface to improve the anti-clotting properties through layer-by-layer assembly technology. New nano-composite scaffolds loaded with vascular stents prepared by tissue engineering technology were used as substitutes for bone matrices, with good biocompatibility and mechanical properties.

## 2. Results and Discussion

### 2.1. Construction and Properties of Vascular Stents

**Figure 1:**
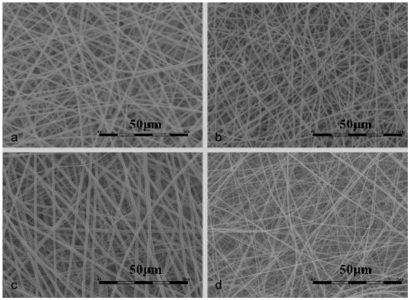
Scanning Electron Microscopy (SEM) images of different vascular stents: (**a**) poly (lactide-ɛ-caprolactone) (PLCL) group; (**b**) PLCL/nano-hydroxyapatite (nHA) group; (**c**) PLCL/collagen (Col)/nHA I group; (**d**) PLCL/Col/nHA II group.

The surface morphology of the vascular stents was observed by scanning electron microscopy (SEM) (Figure 1), exhibiting a highly uniform thickness and ordered arrangement of nano-fibers. Each group of components and concentration is shown in Table 1. The concentration of solution greatly affected the morphology and diameter of electrospun fibers and had to exceed a critical value of electrospinning to carry out the process of electrostatic spinning smoothly. When the solution is diluted, it cannot form a stable fluid and will directly contract under the effects of surface tension and form liquid beads or silk. Mutual entanglement of polymer chains with increasing concentration prevents the deformation of small streams, forming uniform fibers, but if the concentration continues to increase, the solution will be gelatinous and not conducive to formation of neat fibers [31]. In this study, the viscosity of the spinning solution increased significantly with increasing PLCL concentration. nHA had less influence on the fiber diameter than collagen at low content. 0.3% collagen could make the stents exhibit better hydrophilicity and biocompatibility compared with 0.2%, 0.4% and 0.5%, which had been studied and proved by comparative pre-studies.

**Figure 2:**
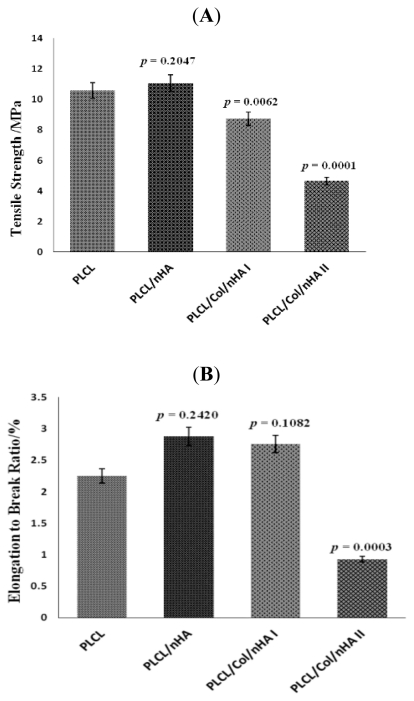
The mechanical properties of different vascular stents evaluated through tensile strength (**A**) and elongation to break ratio (**B**).

Compared with pure PLCL, the tensile strength of stents was improved slightly with the joining of nHA (*p* > 0.05) but it was significantly reduced through addition of collagen (*p* < 0.05) (Figure 2A). The break elongation ratio showed little change in PLCL/nHA group and PLCL/Col/nHA I group compared with pure PLCL group (*p* > 0.05). When the collagen content of scaffolds was higher than the PLCL content, both the tensile strength and breaking elongation ratio were greatly reduced (*p* < 0.01) (Figure 2B).

**Figure 3:**
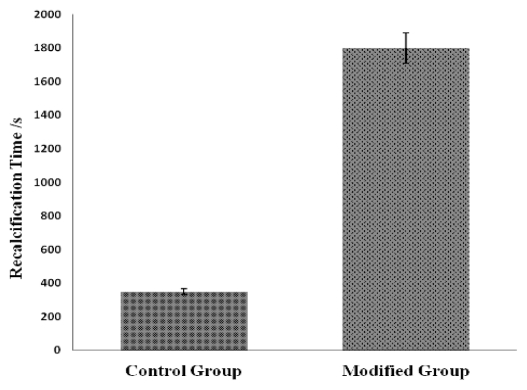
The anticoagulant properties of the stents assessed through recalcification time.

In this experiment, the clotting time of the pure PLCL fiber membrane was about 350 s, while the PLCL/Col/nHA II fiber membrane modified through the electrostatic self-assembly technology showed a clotting time far greater than 1800 s (Figure 3) (all *p* values compared with control group are less than 0.05). Regardless of whether the outermost modified layers were chitosan (CTS) or heparin sodium (HS), all showed good anticoagulant properties, which may have resulted from the existence of a certain amount of HS on the fiber membrane surfaces, interspersed with the outermost layer. HS has good anticoagulant performance so membranes showed a corresponding improvement in performance with increasing HS.

### 2.2. Preparation and Characterization of New Nano-Composite Scaffolds Loaded with Vascular Stents

**Figure 4:**
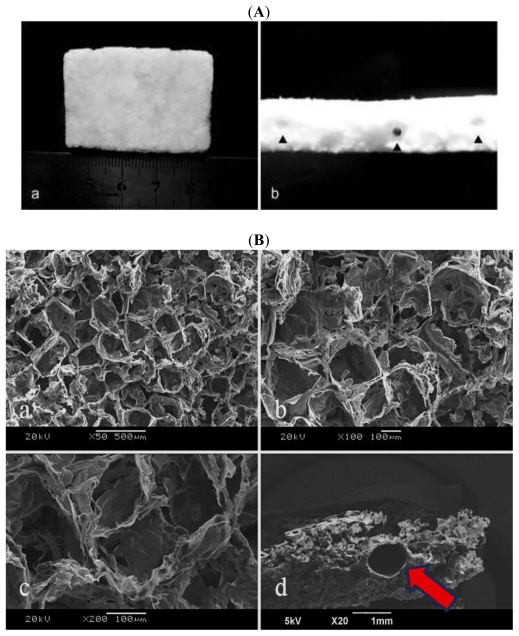
The surface morphology of nano-composite scaffolds observed by naked eye (**A**) and SEM (**B**).

The general appearance of nano-composite scaffolds is white, and porous, with a highly uniform thickness and rough texture (Figure 4A(a)). The small stents were embedded in the nano-composite scaffolds and can be viewed from the schematic diagram (Figure 4A(b) black arrows instruction), the diameter and thickness of which are about 1 mm and 100 μm.

The nano-composite scaffolds showed a porous structure, with a highly uniform pore distribution. The pores were well-connected, as could be seen after the images were magnified in Figure 4B(a,b,c). The pore size was 220~300 μm. SEM images of the small stents embedded in the nano-composite scaffolds can be viewed in Figure 4B(d) (red arrows instruction).

**Figure 5:**
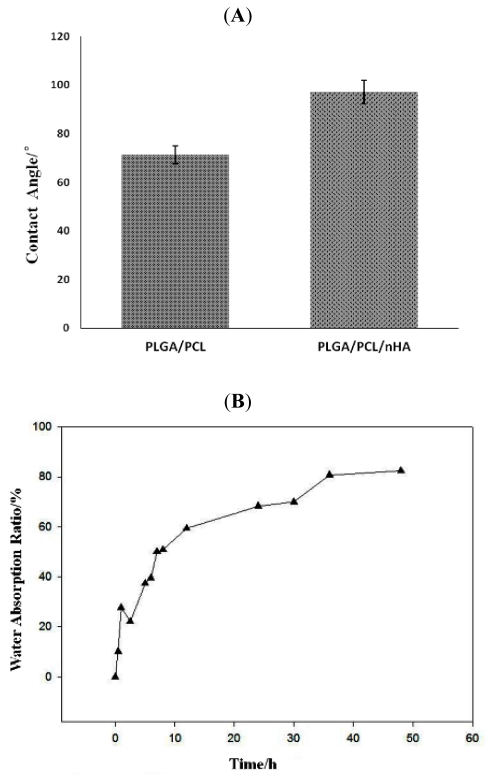
Hydrophilic performance of nano-composite scaffolds assessed by contact angle tests and water absorption experiments.

In the contact angle tests, the contact angle of PLGA/PCL/nHA group slightly increased than PLGA/PCL group (all *p* values compared with no nHA are larger than 0.05) that may be due to nano-scale effect (Figure 5A). In water absorption experiments, the amount of water absorption by the nano-composite scaffolds (PLGA/PCL/nHA) exhibited rapid growth in the first 12 h, followed by a much lower growth rate (Figure 5B).

**Figure 6:**
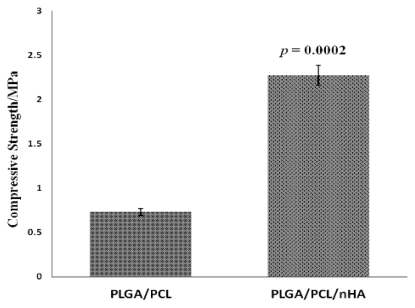
The compressive strength of nano-composite scaffolds.

The compressive strength of nano-composite scaffolds was higher than the control group, which could be seen from Figure 6, demonstrating that strength was increased significantly through addition of nHA (all *p* values compared with no nHA are less than 0.01).

**Figure 7:**
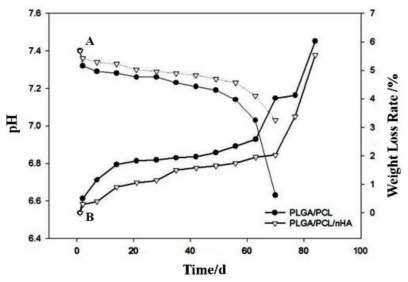
The changes in pH and weight during the process of *in vitro* degradation of nano-composite scaffolds. A represents pH curves and B represents the weight loss curves.

Degradation is an important factor in tissue engineering scaffolds and can impact significantly on cell growth, tissue regeneration and the host response. Therefore, the degradation rate of scaffolds should be synchronized with the cell growth rate. In this study, the pH of scaffolds all decreased with degradation time (Figure 7A), mainly because of the lactic acid produced during the degradation of PLGA and PCL. However, the pH of pure PLGA/PCL decreased more quickly than PLGA/PCL/nHA, perhaps as a result of the neutralization of nHA and lactic acid. Similarly, the weight losses of pure PLGA/PCL were also greater than for PLGA/PCL/nHA (Figure 7B). In addition, the early degradation rates of scaffolds were all relatively slow and accelerated rapidly after 60 h. These results indicate that the PLGA/PCL/nHA scaffolds are more suitable for cell growth and proliferation.

**Figure 8:**
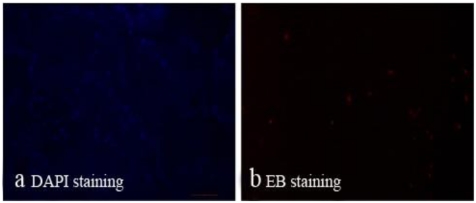
The cell activity of bone marrow mesenchymal stem cells (BMSCs) which seeded on scaffolds of PLGA/PCL/nHA loaded with vascular stents for 7 days was tested by DAPI and EB staining and observed through fluorescence microscopy: blue dots represent living cells (**a**) and dead cells were red (**b**).

From Figure 8a,b, we could see a large number of living cells lived within the scaffolds and only a small amount of apoptosis. These results showed that the scaffolds possess good biocompatibility.

**Figure 9:**
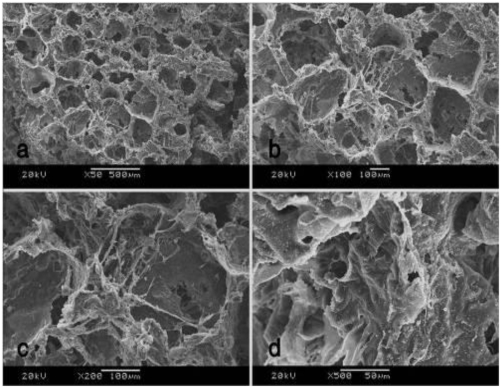
The changes in surface morphology of nano-composite scaffolds seeded with BMSCs after 7 days. SEM images of different magnifications: (**a**) ×50; (**b**) ×100; (**c**) ×200; (**d**) ×500.

From Figure 9a–d, it could see that scaffolds co-cultured with BMSCs after 7 days showed degradation in some areas, while cells exhibited monolayer attachment on the surface of scaffolds. Hydroxyapatite crystals appeared on the surface and inner wall of the scaffolds.

**Figure 10:**
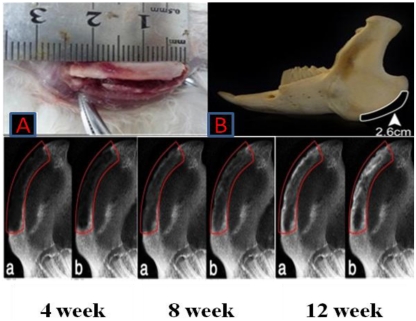
X-ray films of bone regeneration after 4, 8, 12 weeks of implanting operation. **a**,**b** represents PLGA/PCL/nHA loaded with vascular stents group and BMSCs/PLGA/PCL/nHA loaded with vascular stents group respectively. Note: better bone formation and earlier mineralization in group **b** than group **a**.

At 4 weeks after surgery, the shadow areas of low-density in bone defects of PLGA/PCL/nHA loaded with vascular stents group had little changes compared with BMSCs/PLGA/PCL/nHA loaded with vascular stents group, which only slightly increased in the edges of defect areas (as Figure 10 red frame instruction). After 8 weeks, increasing mineralization in the implants was observed, and group b mandibles demonstrated greater new bone formation than the other group. At 12 weeks after implantation, the radio density in bone defects of b group had closed to the surrounding normal bones especially in the edges of bone defects areas. Although, in the middle of the bone defects, the density was not uniform, that has demonstrated successful bone regeneration.

## 3. Experimental Section

### 3.1. Isolation and Culture of BMSCs

New Zealand rabbits’ bone marrow mesenchymal stem cells (BMSCs) were isolated from the tibiae by bone marrow washing combined with density gradient centrifugation, suspended in Dulbecco’s Modified Eagle Medium (DMEM) with 10% fetal bovine serum (FBS) (Gibco BRL), 100 U/mL penicillin and 100 μg/mL streptomycin, and incubated at 37 °C with 5% humid CO_2_. The procedure was carried out as the method described by Hu *et al*. [32]. Cells reached 80% confluence in 8~10 days and then were suspended for passage. Cells of passage 3 were used to seed on scaffolds and for experiments *in vitro* and *in vivo*. Media was changed every 3 days.

### 3.2. Vascular Stent and Nano-Composite Scaffold Preparation

Under room temperature, PLCL (M_w_, 246524) was dissolved in hexafluoro isopropyl alcohol with magnetic stirring. Collagen and nHA [33] which were produced by institute of Nano- and Bio-Polymeric Materials of School of Materials Science and Engineering in Tongji University were then added, with stirring to uniformity by magnetic stirrer and ultrasonication for 5 min. The above prepared solution was injected by a 20 mL syringe, fixed on a syringe pump. The metal needle of the syringe was connected to the anode of a high voltage power supply, the outer diameter of which was 1.2 mm. The receiver was a motor or foil with a rotating shaft located about 17 cm from the needle. The flow rate was then set to 2 mL/h, the voltage controlled to about 17 kV and the speed of the motor at 500 rpm/min. After approximately 2.5 h of electrospinning, the tubular stents were prepared and dried for 24 h under vacuum at room temperature.

In addition, PLGA and PCL (mass ratio 7:3) were dissolved in chloroform solution, with magnetic stirring for complete dissolution to a uniform polymer solution. nHA was added to the solution and dispersed evenly through ultrasonication. Sodium chloride particle sizes of 224~300 μm were obtained by a standard sieve and added to the above polymer solution, and stirred with a glass stirrer to form a uniform paste containing pore-forming agents and with a specific mobility. The paste mixture was then poured into the dish and a polymer film obtained after solvent evaporation, which was then dried in a vacuum oven and cut to appropriate sizes. The shredded polymer film and vascular stent (diameter of 0.9 mm) were placed in a mold, then placed into a hot-press at 6~7 MPa pressure at 70 °C for 3 min. Finally, the solution was tested with silver nitrate until no white precipitate was observed, and the vascular stent support rods were removed and dried.

### 3.3. Characterization

#### 3.3.1. Morphology

The morphology of the vascular stents, nano-composite scaffolds and complexes of cells and scaffolds were studied by SEM (Hitachi S-2360N). All specimens were fixed by 2.5% glutaraldehyde, dehydrated with graded ethanol, dried at the critical point and the surface coated with gold before SEM observation. 3.3.2. Cell Activity The cell activity of bone marrow mesenchymal stem cells (BMSCs) which were seeded onto scaffolds of PLGA/PCL/nHA loaded with vascular stents for 7 days was tested by DAPI and EB staining. The samples of scaffolds seeded BMSCs were added 100 μL DAPI and EB staining solution and incubated at 37 °C for 15 min. Then fluorescence microscopy was used to observe the morphology of living and dead cells at the wavelength conditions of 490 nm and 545 nm respectively. The cell activity was tested by calculating the percentage of living cells of the total cells.

#### 3.3.3. Hydrophilic Performance

The hydrophilic performances of nano-composite scaffolds were measured by detection of the contact angle and water absorption ability. The samples were prepared as polymer films and contact angles were measured by the sessile drop method. In addition, specimens with dimensions 10 × 10 × 20 mm were dried and weighed as W_1_, then immersed and soaked in deionized water at 25 °C. Subsequently, the specimens were removed from the water at specified times of 1, 2, 4, 8, 12, 24 and 48 h, the excess water on the surface of the scaffold was blotted with filter paper and the samples then weighed as W_2_. The water absorption ratio, a, of specimens at different times is defined as:

(1) $$\text{a}=({\text{W}}_{2}-{\text{W}}_{1})/{\text{W}}_{1}\times 100\%$$

#### 3.3.4. Mechanical Properties

The tensile strength of vascular stents and the compressive strength of nano-composite scaffolds were evaluated using a computer controlled tension tester (TUV CMT6104) and pressure tester (DXLL-5000). The tension tester tested the tensile strength of fiber membrane samples with dimensions 80 × 10 mm, which represented the vascular stents. The effective distance was 20 mm and the test speed was 10 mm/min. In addition, the compressive strength of round, flaky specimens with dimensions 15 mm (diameter) × 3 mm (thickness) was evaluated according to the national test standards of China for compression of rigid cellular plastics (GB 8813-1988). The testing was carried out under the following conditions: room temperature, dry and a loading speed of 1 mm/min. Compressive strength was calculated as follows:

(2) σc=P/A

where σc represents the compressive strength; *p* is the load at 30% stent compression; and A is the cross-sectional area (mm^2^).

#### 3.3.5. Anticoagulant Property

(1) Solution configuration: Polyetherimide (PEI) solution, 1 mg/mL; HOAc-NaOAc buffer solution, pH 3.8; HS and CTS solution, 1 mg/mL; (2) Stent surface activation: Stents were immersed in PEI solution for 10 min, removed, rinsed with distilled water and dried; (3) Stent anticoagulation modification: First, stents were immersed in HS solution for 20 min, removed, washed with distilled water and dried; then they were immersed in the CTS solution for 20 min and the above steps repeated 10 times to modify alternately with heparin and chitosan in the outermost layer. Afterwards, fresh Ca^2+^-depleted plasma, 0.025 mol/L CaCl_2_ and fiber membranes were placed into an incubator at 37 °C for 10 min and then removed; 20 μL of the plasma and 20 μL of the CaCl_2_ solution were absorbed by pipette successively and dripped onto fiber membrane, while stirring the plasma with a small glass needle and recording the times of CaCl_2_ solution addition and production of filamentous fibers. The time of anti-clotting recalcification was measured by time interval.

#### 3.3.6. Degradability

Specimens with a dimension of 8 mm (diameter) × 2 mm (thickness) were accurately weighed and placed into numbered test tubes. Then 10 mL of PBS buffer solution were added to the tubes and they were placed into a constant temperature bath at 37 °C. The phosphate buffered saline (PBS) buffer solution was changed once a day for the first two days and then changed weekly, and the pH value recorded with time. Corresponding samples were removed at 1, 2, 4, 7, 14, 21, 28, 35, 42, 49, 56, 63, and 70 days, and the rate of weight loss of the scaffolds and the changes in pH measured with degradation time.

#### 3.3.7. Preparation of the Bone Defect Model and Implantation of Experimental Material

Thirty healthy New Zealand white rabbits weighing about 2.5 kg each were divided into two groups: group a (*n* = 15): PLGA/PCL/nHA loaded with vascular stents group (served as control). Group b (*n* = 15): BMSCs/PLGA/PCL/nHA loaded with vascular stents group. The rabbits were anesthetized with pentobarbital sodium (0.2 mL/kg). A 3 cm incision was made on the bilateral jaw of the rabbits. A critical size defect of 26 × 5 × 3 mm^3^ was made in a buccolingual direction on both sides of the mandible. The same size porous scaffolds or scaffold/cell constructs were inserted into the defects (Figure 10A,B). Five rabbits randomly selected from each group were sacrificed at 4, 8, and 12 weeks after implantation, respectively. Their mandibles were harvested and processed for X-ray detection.

#### 3.3.8. X-ray Detection

After sacrifice, lateral radiographs of the mandibles were taken with the mandibles positioned at 10 cm from the X-ray tube. The X-ray unit (Gendex DEN S-O-MAT, Milano, IL, USA) was set at 70 kV and 7 mA with a 0.26 s exposure time.

#### 3.3.9. Statistical Analysis

All data were expressed as means ± SD (standard deviation). One-way analysis of variance (ANOVA) and Student’s *t* test were conducted to compare differences between groups using SPSS 14.0 [34]. Differences were considered to be significant when *p* < 0.05 or *p* < 0.01.

## 4. Conclusions

PLCL/Col/nHA vascular stents were successfully prepared by electrospinning and the surface of the stents modified by PEI, heparin and chitosan. The stents can be used in tissue engineering as small vessel stents with good mechanical, biological and anticoagulant properties. In addition, new nano-composite PLGA/PCL/nHA scaffolds loaded with the PLCL/Col/nHA vascular stents were prepared by a thermoforming-particle leaching method. These can serve as ideal scaffolds for tissue engineering with good mechanical, biological and osteogenic properties for related experimental studies of bone tissue engineering both *in vivo* and *in vitro*.

## Figures and Tables

**Table 1:** The components of each group.

Group	Components
PLCL	PLCL, 10% (g/mL)
PLCL/nHA	PLCL, 10% (g/mL) + nHA, 0.03% (g/mL)
PLCL/Col/nHA I	PLCL, 10% (g/mL) + Col, 0.3% (g/mL) + nHA, 0.03% (g/mL)
PLCL/Col/nHA II	PLCL + Col + nHA, 0.07% (g/mL), PLCL/Col = 1:2 (g/g)
